# Abortion of Small-Pox

**Published:** 1895-03

**Authors:** 


### Progress in Medicine.

### Abortion of Small-Pox.

THE LATEST DISCOVERY.—Our readers will doubtless recall Dr. T. C. Osborn's article in the *Texas Sanitarian* on the abortion of small-pox after the fever had set in; the arrest, as he claimed, of the disease; the prevention of eruption and consequent pustulation and *ergo* the prevention of pitting. Also the TEXAS MEDICAL JOURNAL'S remarks on it at the time. Dr. Osborn used a strong solution of corrosive sublimate all over the body, and claims that it destroyed the colonies of bacteria in situ.

It is believed now that the disease can be aborted by the injection of serum of an immune animal, and experiments are being made at Washington in that direction. The following we take from the U. S. Government Abstracts of Sanitary Reports. It will be read with interest:

"Just previous to the reappearance of small-pox in this city I had made arrangements with Dr. Ralph Walsh, proprietor of the national vaccine farm, to conduct a line of inquiry concerning the nature of vaccinia, and, while engaged in this, took advantage of the cases of small-pox to put a theory into effect. It has been already demonstrated by Maurice Raynaud and Sternberg that the blood serum of an immune animal destroys the potency of vaccine lymph. It has occurred to me, as well as to others, that this fact could be utilized in the treatment of small-pox by the injection of this serum in patients suffering with the disease.

"Accordingly, on December 23, 1894, I took a liter of blood from a heifer calf which had been previously vaccinated on November 26. At the time of bleeding, the local effects of the vaccination had disappeared, the animal to all appearances was sound and well. As soon as the blood was withdrawn it was taken to the laboratory, where on the next day, about 350 c. c. of tolerably clear serum were drawn off.

"A part of the serum was transferred to a small sterilized flask, while another part of equal quantity was passed through a special filter in order to remove the blood corpuscles and any chance bacteria which might have contaminated it.

"About 35 minims of pure vaccine lymph (two days old) was added to 2 c. c. of the filtered and unfiltered serum respectively. After a few hours' exposure, the serum was sent out to the vaccine farm, and a small heifer was inoculated in the usual manner with each sample. The results were negative in both instances, demonstrating that the process of filtration does not affect its power. The substance which possesses this neutralizing power is soluble, and not confined to the corpuscular elements.

"Accordingly I prepared a considerable quantity of this filtered serum and sent it to Dr. Elliot, the physician in charge of the small-pox hospital, accompanied with the request that he would use this serum upon such cases of variola as were in his judgment suitable for the experiment. It was suggested that the treatment be given to fresh cases before the stage of pustulation, for these I thought would react more favorably to the serum than older cases.

"As a trial dose 15 c. c. was suggested, to be repeated within from eight to ten hours if there was no reaction or amelioration of the symptoms. It was, however, the opinion that a larger dosage would have to be given before such effects would be noticed.

"It was suggested that a careful note should be made of the patient's condition before the administration of the serum, and accurate observations be made of the pulse, respiration, temperature, the presence or absence of albumen in the urine, and the condition in the eruption. It is much to be regretted by Dr. Elliot and myself that an opportunity did not offer to give serum to cases in the first stage of the eruption, whereas the treatment was confined to two which were in the pustular stage.

"Being in telephonic communication with Dr. Elliot, we managed to discuss the cases from day to day, and make mutual suggestions as to the modification of treatment. Dr. Elliot kindly sent the notes of the cases under treatment and embodies therein his conclusions, drawn from his observations on the effects of the serum."

[Dr. Elliot's report is omitted. One case died, and the other was thoughtt o be modified by the treatment.—ED.]

"From the history of the two cases treated with the serum, it appears that it does have a modifying effect upon the disease, especially upon the eruption (Case II).

"I am informed by Dr. Elliot that it was his belief that by administering the serum to the first case, life was prolonged at least seventy-two hours.

"Since it appears possible to modify the pustular stage of smallpox, and in this case have little or no pitting follow, it certainly appears reasonable to assume that it would have even yet a greater power over the disease in its first stages.

"Since it seems possible to mitigate the attack of variola, it also appears rational to presume that the serum would have power to render susceptible persons refractory to the disease. It is intended to pursue my investigations on these lines and incorporate the results of my experiments in a forthcoming commuication.

"Very respectfully submitted,

"J. J. KINYOUN,

"Passed Assistant Surgeon, M. H. S."

And as germane to the subject, we reproduce the following from Dr. Murfee's new *Journal of Health, Sanitation and Climatology of the Southern States*, published at Washington, and mention of which will be found elsewhere in this issue:

"Up to this time the researches of bacteriology have been of more benefit to preventive medicine than to therapeutics. Only in a very limited measure has the knowledge of a specific cause of disease been conducive to specific treatment. The latest discoveries of bacteriologists, among whom is our distinguished fellow member, Col. George M. Sternberg, Surgeon-General United States Army, seem to indicate a new departure in the treatment of infectious diseases.

"Although as sanitarians we are more directly interested in preventive than curative medicine, yet the subjects are so closely allied that progress in one is a step toward the other. In this connection we deem it appropriate to reproduce the following extract from the masterly address, "On General Medicine," read at the Detroit meeting of the American Medical Association, by our esteemed friend, Dr. A. L. Gihon, Medical Director United States Navy: 'Erhlich's experiments with pathogenic toxalbumins, Sewall's, showing that immunity from poisoning by rattlesnake venom may be produced by small doses of its toxic agent, and other facts, give strong support to the view that all infectious diseases are due to the action of substances resembling the toxalbumins already discovered, and that acquired immunity from any of them is due to the formation of anti-toxines in the blood of the immuned animal.'

"Sternberg suggests the possibility that in these diseases the toxalbumin, which gives them their specific character, is a product of the living cells of the body of the infected individual, and says the inference is justifiable that the blood of tissue-juices of any individual who has recently suffered an attack of small-pox and scarlet fever contains an anti-toxine which would neutralize the active poison in the circulation of another person immediately after infection.

"Dr. Sternberg holds the experiment warrantable to ascertain whether a small quantity of blood drawn from the veins of the protected person would suffice to arrest or modify the course of these diseases. The transfusion of a moderate amount of such blood might be curative, or confer immunity in advance of infection, and possibly an anti-toxine may be obtained from the blood of vaccinated calves, which would have a curative action on small-pox.

"Dr. Sternberg has himself demonstrated, by recent experiments with the blood of vaccinated calves, that there is something in the blood which does neutralize the specific virulence of vaccine virus, both bovine and humanized.

"These experiments are still in their infancy; it is impossible to foresee the results they may lead to. A new light seems to to be dawning."

